# Complete mitochondrial genome and the phylogenetic position of the blackspotted catshark *Halaelurus burgeri* (Carcharhiniformes: Scyliorhinidae)

**DOI:** 10.1080/23802359.2016.1168722

**Published:** 2016-06-20

**Authors:** Hao Chen, Wenyong Ding, Lezhou Shan, Xiao Chen, Weiming Ai

**Affiliations:** a Department of Marine Biotechnology, School of Life Science, Wenzhou Medical University, Wenzhou, Zhejiang, PR China;; b Zhejiang Mariculture Research Institute, Wenzhou, Zhejiang, PR China

**Keywords:** *Halaelurus buergeri*, mitochondrial genome, Scyliorhinidae

## Abstract

In this study, the mitogenome of the blackspotted catshark *Halaelurus burgeri* was first determined. This circle molecular was rich in A (30.7%)+T (30.4%), poor in C (25.0%)+G (13.8%) and contained 13 protein-coding genes, 2 rRNA genes, 22 tRNA genes and a longest control region (3481 bp with lots of repeated motifs) in sharks. Two start codons (ATG and GTG) and two stop codons (TAG and TAA/T) were found in the protein-coding genes. The 22 tRNA genes ranged from 66 bp (tRNA-*Ser*2) to 75 bp (tRNA-*Leu*1). The phylogenetic result showed that *H. burgeri* did not cluster to the two species of Scyliorhinidae (*Cephaloscyllium umbratile* and *Scyliorhinus canicula*).

Often found on the continental shelf and sublittoral zone above 100 m, the blackspotted catshark *Halaelurus burgeri* (Carcharhiniformes: Scyliorhinidae), a kind of oviparous shark, was mainly distributed in Northwest Pacific but collected less than a degree west of the northwestern boundary of the Western Central Pacific (Compagno 1984; Yamada et al. 1995; Compagno & Niem 1998). In this study, we first sequenced the complete mitogenome of *H. burgeri* and uploaded it to Genbank, then analyzed the phylogenetic position of this species.

One specimen of *H. buergeri* was captured in Taiwan Strait, preserved in the Museum of Marine Biology in Wenzhou Medical University with voucher DS2011052443. The experimental protocol and data analysis methods followed Chen et al. (2014). Including *H. buergeri*, 29 species of Carcharhiniformes with complete mitogenomes available in the GenBank were selected to construct the phylogenetic tree by Bayesian method, fulfilled with the GTR + I + G model and using three partitions: 12S and 16S rRNA genes, the first and second codons of the 12 protein-coding genes (except *ND*6 gene).

The mitogenome of *H. buergeri* (Genbank accession no. KU892589) reaches 19,100 bp in length, which is longer than other reported mitogenomes of sharks. However, its gene arrangement and transcriptional orientation are identical to most mitogenomes of vertebrates, and its nucleotide base composition is also rich in A (30.7%) + T (30.4%), poor in C (25.0%) + G (13.8%) as most sharks’ mitogenomes. This genome owns 13 protein-coding genes, of which one gene (*ND*6) is in light strand, whereas others are in heavy strand. Except two genes (*CO*1 and *ND*4) using codon GTG as started codon, the other genes all starts with standard ATG codon. While as to the terminal codon, all protein-coding genes uses two typical terminal codons TAG and TAA/T. Both 12S rRNA (958 bp) and 16S rRNA (1665 bp) genes are between tRNA-*Phe* and tRNA-*Leu*1 genes, separated by tRNA-*Val* gene. All tRNA genes except tRNA-*Ser*2 can fold into a typical clover-leaf secondary structure, and the length of them ranges from 66 bp (tRNA-*Cys*) to 75bp (tRNA-*Leu*1). To our knowledge, the control region (3481 bp) of *H. Buergeri* is the longest one in sharks. A 60 bp tandem repeat motif (TRM) is found in the control region repeated 27 times near the tRNA-*Pro* gene, and followed by a 47 bp TRM repeated nine times. It is the reason why this control region was much longer than the normal level.

Most nodes of the Bayesian tree are well supported. The tree shows six families of the order Carcharhiniformes (Figure 1). Among these families, the family Scyliorhinidae is relatively primitive and basal as the tree displayed. However, *H. burgeri* clusters to the remaining five families with 100% BI value instead of two species of Scyliorhinidae (*Cephaloscyllium umbratile* and *Scyliorhinus canicula*). It suggests that the family Scyliorhinidae is a polyphyletic group.

**Figure 1.**
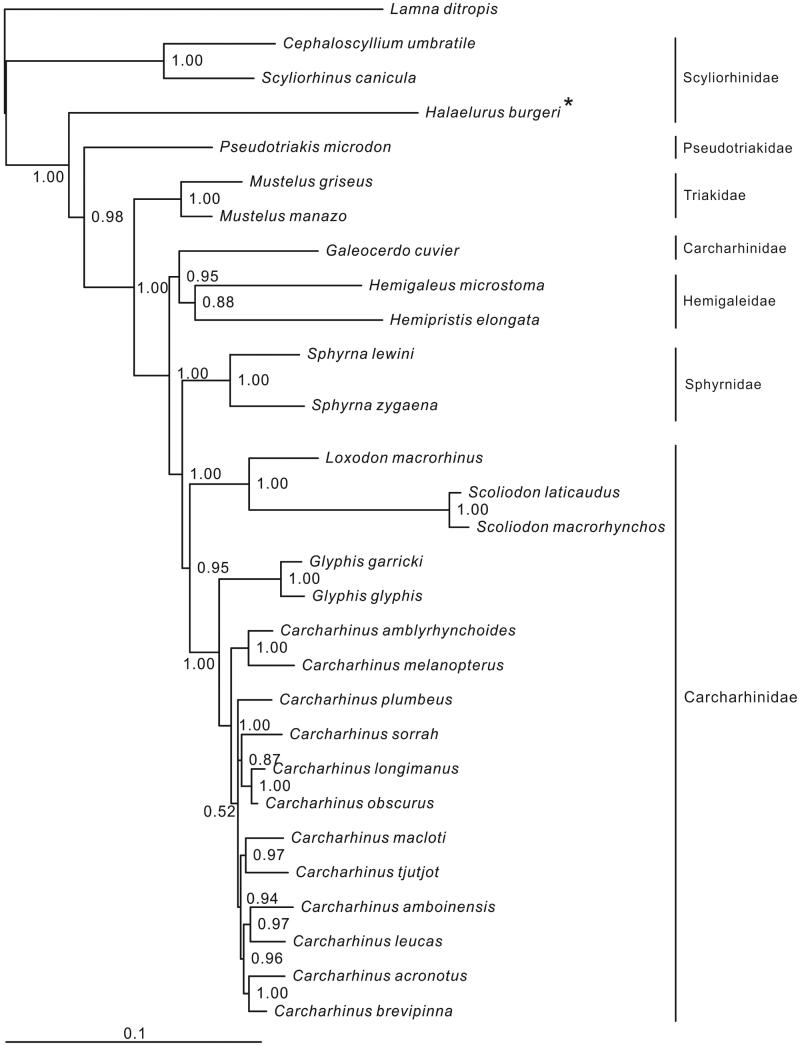
Phylogenetic position of *Halaelurus burger Lamna ditropis* (KF962053.1) was selected as the out group. The 29 species from the order Carcharhiniformes were: *Carcharhinus acronotus* (NC_024055.1), *C. amblyrhynchoides* (NC_023948.1), *C. amboinensis* (NC_026696.1), *C. brevipinna* (KM244770.1), *C. leucas* (KF646785.1), *C. longimanus* (NC_025520.1), *C. macloti* (NC_024862.1), *C. melanopterus* (NC_024284.1), *C. obscurus* (NC_020611.1), *C. plumbeus* (NC_024596.1), *C. sorrah* (NC_023521.1), *C. tjutjot* (KP091436.1) *Galeocerdo cuvier* (NC_022193.1), *Loxodon macrorhinus* (KT347599), *Scoliodon laticaudus* (KP336547.1)*, S. macrorhynchos* (NC_018052.1), *Glyphis glyphis* (NC_021768.2), *G. garricki* (KF646786.1), *Mustelus griseus* (NC_023527.1), *M. manazo* (NC_000890.1), *Cephaloscyllium umbratile* (KT003686), *Halaelurus burgeri* (KU892589), *Hemigaleus microstoma* (KT003687), *Hemipristis elongata* (KU508621), *Scyliorhinus canicula* (NC_001950.1), *Pseudotriakis microdon* (NC_022735.1), *Sphyrna lewini* (NC_022679.1) and *Sphyrna zygaena* (NC_025778.1).
